# Retraction: Does green investment affect environment pollution: Evidence from asymmetric ARDL approach?

**DOI:** 10.1371/journal.pone.0323219

**Published:** 2025-05-07

**Authors:** The PLOS One Editors

The *PLOS One* Editors retract this article [1] due to concerns about potential manipulation of the publication process and authorship. These concerns call into question the validity and provenance of the reported results. We regret that the issues were not identified prior to the article’s publication.

Before the above concerns were identified, the first author (YS) requested to correct their affiliation to School of Economics and Management, Southwest Petroleum University, Chengdu, China.

YS agreed with the retraction. MSM responded but expressed neither agreement nor disagreement with the editorial decision. SUR, NSH, and MSEA either did not respond directly or could not be reached.
